# Correlation of Intra-Tumor ^18^F-FDG Uptake Heterogeneity Indices with Perfusion CT Derived Parameters in Colorectal Cancer

**DOI:** 10.1371/journal.pone.0099567

**Published:** 2014-06-13

**Authors:** Florent Tixier, Ashley M. Groves, Vicky Goh, Mathieu Hatt, Pierre Ingrand, Catherine Cheze Le Rest, Dimitris Visvikis

**Affiliations:** 1 INSERM, UMR1101, LaTIM, CHRU Morvan, Brest, France; 2 Institute of Nuclear Medicine, UCL, Euston Road, London, United Kingdom; 3 Division of Imaging Sciences and Biomedical Engineering, Kings College London, St Thomas Hospital, London, United Kingdom; 4 Epidemiology & Biostatistics, CIC Inserm 1402, CHU Milétrie, Poitiers, France; 5 Department of Nuclear Medicine, CHU Miletrie, Poitiers, France; The University of Chicago, United States of America

## Abstract

Application of textural features analysis to 18F-fluorodeoxyglucose (^18^F-FDG) positron emission tomography (PET) images has been used to characterize intra-tumor uptake heterogeneity and has been shown to reflect disease outcome. A current hypothesis is that ^18^F-FDG uptake heterogeneity may reflect the physiological tracer uptake related to tumor perfusion. The purpose of our study was to investigate the correlations between intra-tumor uptake heterogeneity and vascular parameters derived from dynamic contrast enhanced (DCE) computed tomography (CT) obtained from an integrated ^18^F-FDG PET/perfusion CT examination.

**Methods:**

Thirty patients with proven colorectal cancer prospectively underwent integrated ^18^F-FDG PET/DCE-CT to assess the metabolic-flow phenotype. Both CT blood flow parametric maps and PET images were analyzed. Correlations between PET heterogeneity and perfusion CT were assessed by Spearman's rank correlation analysis.

**Results:**

Blood flow visualization provided by DCE-CT images was significantly correlated with ^18^F-FDG PET metabolically active tumor volume as well as with uptake heterogeneity for patients with stage III/IV tumors (|ρ|:0.66 to 0.78; p-value<0.02).

**Conclusion:**

The positive correlation found with tumor blood flow indicates that intra-tumor heterogeneity of ^18^F-FDG PET accumulation reflects to some extent tracer distribution and consequently indicates that ^18^F-FDG PET intra-tumor heterogeneity may be associated with physiological processes such as tumor vascularization.

## Introduction

Colorectal cancer is associated with high morbidity, with a 5-year survival of below 50% for rectal cancer [1]. The role of ^18^F-FDG PET/CT is well established in this cancer model for the detection of recurrent and residual disease as well as in pre-operative staging [2]. In current clinical practice, clinico-pathological staging is used to identify patients who may benefit from neoadjuvant chemo-radiation pre-operatively and adjuvant chemotherapy following surgery [3}. However ^18^F-FDG PET is becoming an increasingly established imaging modality in colorectal cancer for staging and response assessment [3], [4]. The prognostic and/or predictive value of PET derived parameters with regard to survival or early assessment of response to therapy (during or before treatment), has been the focus of several studies [5]–[7]. For instance, extraction of parameters with significant predictive value from the baseline ^18^F-FDG PET scan has been proposed using metabolically active tumor volumes (MATV, the functional volume of the tumor as it can be seen and delineated on an ^18^F-FDG PET image) [8].

On the other hand, there has recently been increasing interest in the assessment of intra-tumor ^18^F-FDG heterogeneity, demonstrating an association between such heterogeneity measures on baseline ^18^F-FDG PET images and overall patient outcome [9]–. Such studies have been performed within the context of locally advanced esophageal cancer [9], lung cancer [10], as well as cervix and head and neck cancer [11]. The characterization of intra-tumor uptake heterogeneity can be categorized into global, regional (tumor sub-volumes) and local (a few voxels) scales. It has been hypothesized that the measured ^18^F-FDG PET activity distribution heterogeneity may be correlated with several physiological processes including glucose metabolism but also with necrosis, vascularization and angiogenesis [12], [13].Within this context a robust analysis of the intra-tumor ^18^F-FDG uptake heterogeneity could therefore potentially allow the assessment of such physiological tumor characteristics.

On the other hand, association of ^18^F-FDG uptake heterogeneity with subjacent physiological processes cannot be performed intuitively or visually, because of the complex and high order nature of the involved parameters obtained through texture analysis. Previous CT studies using dynamic contrast enhanced sequences to measure regional blood flow (BF in ml/100 g/min), blood volume (BV in ml/100 g), mean transit time (MTT in secs) and permeability surface (PS in ml/100 g/min) have suggested that this imaging technique highlights physiological vascular information and may provide an *in-vivo* marker of tumor angiogenesis [14] or tumor vascularization [15] and can be useful for monitoring neoadjuvant chemotherapy and radiation therapy [16]. BV is defined as the volume of blood within the vasculature of the tumor. BF is defined as BV rate (per min) through the vasculature in a tumor. BV was shown to be a surrogate marker of microvascular density, which is a measure of angiogenesis and has been shown to be an important prognostic factor in many cancer models [17]. MTT is a measurement of the average time necessary for the blood elements to go through the vasculature. Finally, PS is linked to the diffusion of the contrast agent and consequently quantifies the permeability of the blood vessel tumor barrier.

The main objective of this study is therefore to investigate the correlation between ^18^F-FDG PET derived parameters, including standardized uptake value (SUV) measurements, MATV and several intra-tumor uptake heterogeneity parameters, previously identified as predictive of response to chemo-radiotherapy [9], and dynamic contrast enhanced CT (perfusion CT) based parameters (BF, BV, MTT and PS). This study represents a first attempt to elucidate on the underlying physiological processes associated with measured regional and local ^18^F-FDG intra-tumor heterogeneity provided by texture analysis.

## Materials and Methods

### Patients

The study was approved by the University College London (UCL) ethics committee. A written consent also approved by the UCL ethics committee was used in this study for each enrolled patient. Patients with primary colorectal cancer were recruited prospectively from 2007 to 2010 and were scheduled for surgery. In addition to standard staging examinations, eligible adult patients underwent an additional integrated ^18^F-FDG PET/perfusion CT examination prior to surgery, provided there were no contraindications (uncontrolled diabetes; pregnancy, previous reaction to intravenous contrast agent, renal impairment: serum creatinine>120 µmol/L). The study population consisted of 30 patients (20-male; 10-female; mean age 68±9 y). Staging was performed according to the American Joint Committee on Cancer (AJCC) directives [18]. Seventeen patients had a stage I or II, 13 had a stage III or higher disease. The patients' characteristics are summarized in Table 1.

**Table 1:** Patients characteristics.

Characteristic		No. Of patients (%)
**Sex**		
	Male	20 (67)
	Female	10 (33)
**AJCC stage**		
	I	6 (20)
	II	11 (37)
	IIIb	4 (13)
	IIIc	3 (10)
	IV	6 (20)
**TNM stage**		
	T1	0 (0)
	T2	11 (37)
	T3	16 (53)
	T4	3 (10)
	N0	20 (67)
	N1	7 (23)
	N2	3 (10)
	M0	23 (77)
	M1	7 (23)
**Site**		
	Rectum	10 (30)
	Caecum	7 (23)
	Sigmoid	5 (17)
	Colon	5 (17)
	Rectosigmoid	2 (7)
	Splenic flexure	1 (3)

### ^18^F- FDG PET/CT

All patients underwent combined ^18^F-FDG-PET/perfusion CT on a dedicated integrated PET/64-detector-CT (Discovery VCT, GE Healthcare, Amersham, UK). Fasted patients received an injected dose of (270±80) MBq ^18^F-FDG and imaging performed on average 66+/-7 min after injection. CT for attenuation correction was from the skull base to upper thigh: 140 kV, 40 mAs, pitch 1.5, 3.75-mm detectors, 5-mm collimation. The PET static emission scan covering the same anatomical area was carried out in 3D mode, consisting of an emission scan of 8 minutes/bed position. Transaxial emission images of 5.47×5.47×3.27 mm (in plane matrix size 128×128) were reconstructed using ordered subsets expectation maximization (OSEM) with two iterations and 28 subsets. The axial field of view was 148.75 mm. No motion correction was applied, since motion can be considered negligible in the body region concerned by the colorectal cancer targeted in this study. This was followed immediately by a perfusion CT examination (120 kV, 60 mAs, axial mode, 2-second interval for first 40 seconds, then 5-second interval; total acquisition: 150 seconds; effective dose: 9 mSv; 10 second delay from injection) which was acquired following intravenous iohexol (50 mL, 350 mg/mL iodine Omnipaque, GE Healthcare; Chalfont St Giles,UK; at 5 mL/s).

### Image analysis

#### PET tumor delineation

For each patient, primary tumors were identified on ^18^F-FDG PET images by an experienced nuclear physician. Tumors were then delineated automatically using a previously validated fuzzy locally adaptive Bayesian (FLAB) algorithm [19]. This algorithm allows automatic tumor delineation by computing a probability of belonging to a given “class” (e.g. tumor or background) for each voxel within a 3D region of interest containing the tumor and its surrounding background. This probability is calculated by taking into account the voxel's intensity with respect to the statistical distributions (characterized by their mean and variance) of the voxels in the various regions of the image, as well as its special correlation with neighboring voxels in 3D.

#### PET extraction of quantitative indices

Different parameters were extracted from these baseline PET images using the delineated tumor. These parameters included maximum SUV (SUV_max_), the metabolic active tumor volume (MATV), the tumor lesion glycolysis (TLG) defined as the product of mean SUV and MATV [20], and 4 local and regional tumor heterogeneity parameters derived from texture analysis [9].

The parameters under consideration are listed in Table 2. SUV_max_ provides a global characterization of the uptake radiotracer within the tumors. Textural features analysis on the other hand provides a large number of parameters [9]. These parameters were obtained in two steps. Firstly matrices that describe the relationship between voxels within the tumor delineated volumes were extracted [21], [22] As a second step the derived matrices were used to calculate different texture indices related to the heterogeneity of the intra-tumor activity distribution. In this study, we have considered parameters given by 1) co-occurrence matrices that provide information about heterogeneity at a local scale and 2) intensity size-zone matrices describing homogeneous tumor areas which are able to describe heterogeneity at a regional scale. Only a limited number of texture parameters that have been previously shown to be both reproducible and of potential clinical interest in different cancer models [9], [23], [24], as well as being robust with respect to the overall image spatial resolution and the tumor delineation methodology [25] were considered here. Before the derivation of the texture matrices, the voxels intensity within the delineated volume were resampled into 64 distinct values with the following formula:
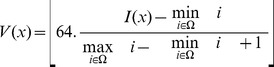
(Eq.1)where, I is the original voxel intensity, Ω is the set of voxels in the delineated volume.

**Table 2:** Texture type, scale and associated features.

Type	Feature	Scale
Features based on intensity histogram	Maximum intensity (SUV_max_)	Global
Features based on intensity size-zone matrix	Size-zone variability (SZV)	Regional
	Intensity variability (IV)	
Features based on co-occurrence matrices	Entropy	Local
	Homogeneity	

The normalization value of 64 was chosen according to previously conducted studies [23], [24] that identified this value as optimal for resampling the typical range of intratumor SUV voxel values encountered (between 2 and 25), providing sufficient sampling range to avoid compromising the reproducibility of the associated texture parameter measurements. The co-occurrence matrices (M) were used to describe relationships between contiguous voxels in a specific angular direction by summing all intensity transitions in the delineated volume. From these matrices we have considered the entropy and the homogeneity defined by:
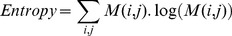
(Eq.2)

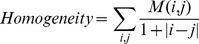
(Eq.3)where, M is a co-occurrence matrix, i,j are the rows and columns index. For each delineation 13 co-occurrences matrices were considered (one for each angular direction) and finally we use as indices the mean of entropy and homogeneity for these 13 directions.

The intensity size-zone matrices (N) were used to describe the relationship between homogeneous areas into the tumor and their intensity. These matrices have 64 lines (the value used for the image discretization) and the number of columns is equal to the number of voxel of the largest homogeneous area found inside the segmented tumor volume. Using these matrices two indices were considered allowing the quantification of intensity variability into the homogeneous areas (IV) and the variability in the size of these homogenous areas (SZV). These indices were calculated using the following formula:
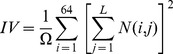
(Eq.4)

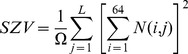
(Eq.5)where, Ω is the number of homogeneous areas within the tumor, L the size of the largest homogeneous area within tumor and N(i,j) the number of areas with intensity i and size j.

**Figure 1:**
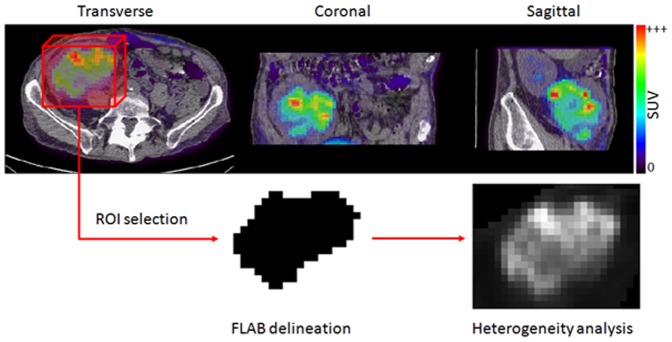
Heterogeneity analysis process. Illustration of heterogeneity analysis process: in upper an example of tumor (PET/CT with transverse, coronal and sagittal view), at the bottom: illustration of the FLAB delineation after a manual ROI selection that allows heterogeneity analysis.

Figure 1 illustrates for a patient example the different steps of the implemented heterogeneity analysis process.

#### Perfusion CT

Arterial perfusion fraction was calculated based on the maximum slope and dual-input-compartment model methods using freely available software (Basama Perfusion Ver. 3.1.0.4) [26]. The perfusion CT acquisition was assessed by an experienced radiologist using commercial software based on distributed parameter analysis (Perfusion 3.0; GE Healthcare, Chalfont St Giles, UK). A processing threshold of -50 to 150HU was applied. A smoothed arterial-time enhancement curve was derived by placing a circular 20 mm^2^ region of interest within the best-visualized artery [27]. From the automatically generated parametric maps, tumor blood flow, blood volume, mean transit time and permeability surface was obtained by defining a tumor region of interest just within the tumor outline on all images where the tumor was visible. The mean value was recorded for each of the four parameters considered.

### Statistical analysis

Statistical analyses were performed using MedCalc software (version 13.0.0.0, MedCalc Software, Belgium). The comparisons between PET and perfusion CT parameters were carried out using Spearman's rank coefficient (ρ), while 95% confidence intervals (CI) for ρ were also obtained. An independent Student t-test was performed to determine the differences between perfusion CT and intra-tumor uptake heterogeneity parameters. The ability to classify patients according to the MATV of their primary tumor with respect to staging was investigated using Mann-Whitney U tests as recommended for small and non-Gaussian distributed samples. P-values under 0.05 were considered significant after the application of Bonferroni correction for multiple comparisons.

## Results

According to the Mann-Whitney U test a strong correlation was found between MATV and stage, as MATVs of early stage tumors (I/II) were significantly smaller than for higher stages (III/IV; 15±10 cm^3^ vs. 31±20 cm^3^, p = 0.006). Table 3 provides Spearman's rank correlation results and their associated p-value between perfusion CT derived parameters (BF, BV, MTT and PS) and PET derived heterogeneity parameters for stage III/IV patients, since for stage I/II none of the investigated parameters were found to be correlated with perfusion indices.

**Table 3:** Spearman's rank correlation coefficient, |p|, between PET and CT perfusion parameters and theirs associated p-values for stage III/IV patients.

	Average PS			Average BF			Average BV			Average MTT			
	|ρ|	p	95% CI	|ρ|	p	95% CI	|ρ|	P	95% CI	|ρ|	P	95% CI	
**IV**	0.14	1	−0.64 to 0.45	**0.75**	**0.02**	**0.35 to 0.92**	0.34	1	−0.26 to 0.75	0.59	0.21	−0.86 to −0.06	**Regional heterogeneity parameters**
**SZV**	0.05	1	−0.52 to 0.59	0.70	0.06	−0.90 to −0.24	0.27	1	−0.71 to 0.33	0.63	0.14	0.12 to 0.88	
**Homogeneity**	0.18	1	−0.67 to 0.41	**0.78**	**0.01**	**0.40 to 0.93**	0.49	0.63	−0.09 to 0.82	0.51	0.56	−0.82 to 0.06	**Local heterogeneity parameters**
**Entropy**	0.03	1	−0.57 to 0.53	0.66	0.10	0.17 to 0.89	0.28	1	−0.32 to 0.72	0.58	0.28	−0.86 to −0.05	
**SUV_max_**	0.03	1	−0.53 to 0.57	**0.29**	**1**	**−0.31 to 0.73**	0.12	1	−0.46 to 0.63	0.30	1	−0.73 to 0.30	**Intensity histogram parameters**
**MATV**	0.04	1	−0.58 to 0.52	0.71	0.05	0.26 to 0.90	0.31	1	−0.29 to 0.74	0.62	0.14	−0.87 to −0.11	**Others parameters**
**TLG**	0.13	1	−0.46 to 0.63	0.50	0.56	−0.07 to 0.82	0.25	1	−0.35 to 0.71	0.44	0.91	−0.80 to 0.15	

**Figure 2:**
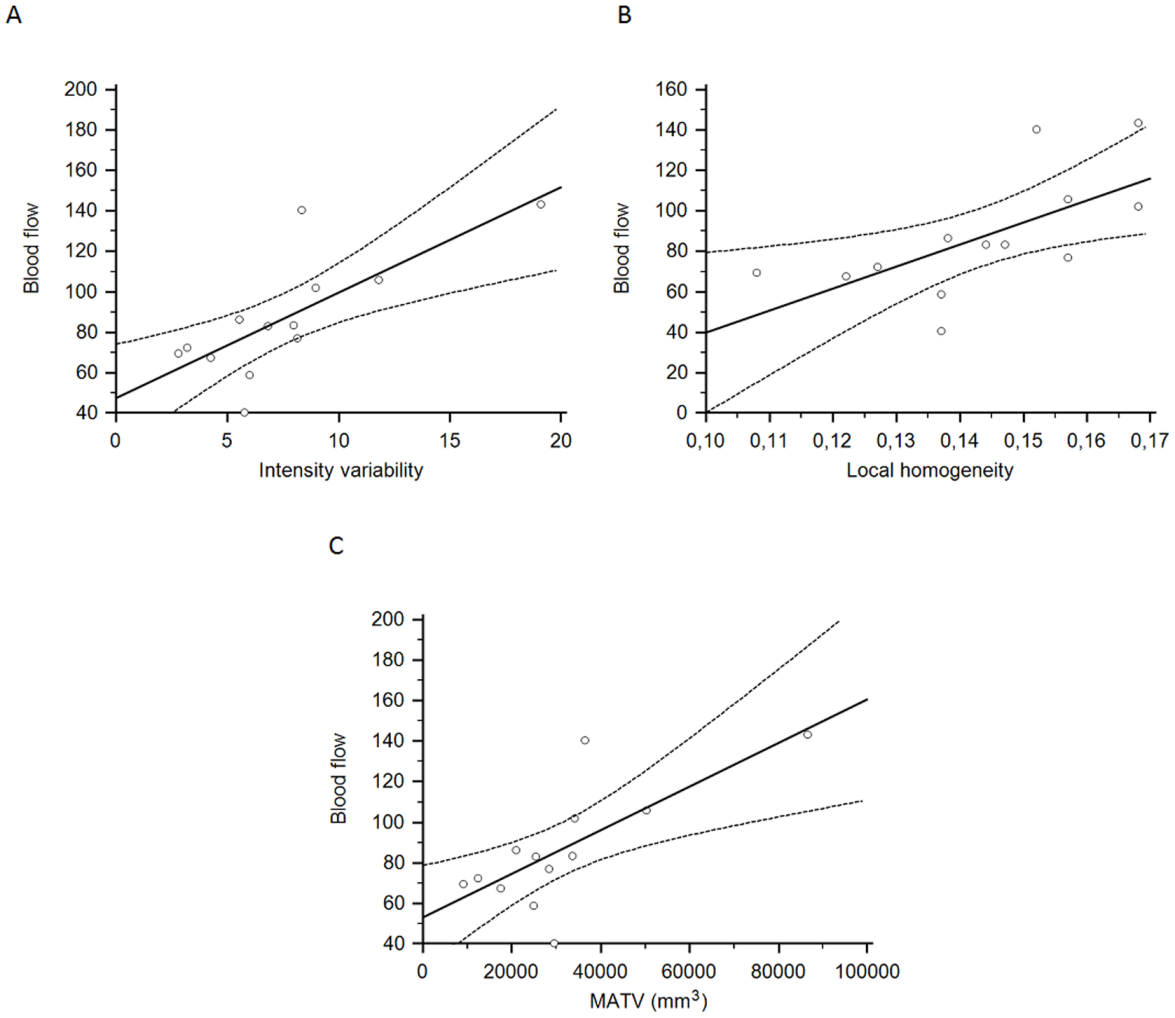
Correlation between PET heterogeneity and BF. Scatter diagrams with regression line (solid line) and associated 95% CI (curves above and below regression line represented upper and lower bounds of 95% CI) showing the correlation of BF with A) IV (|ρ| = 0.75, 95%CI: 0.35 to 0.92, p = 0.02), B) local homogeneity (|ρ| = 0.78, 95%CI: 0.40 to 0.93, p = 0.01), C) MATV (|ρ| = 0.71, 95%CI: 0.26 to 0.90, p = 0.05) in stage III/IV tumors.

For stage III and IV patients, IV, homogeneity and MATV were significantly correlated with BF. For example, the regional intra-tumor intensity variability was associated with a Spearman's rank correlation of 0.75 (95% CI: 0.35 to 0.92, p = 0.02), whereas the local homogeneity was associated with a Spearman's rank correlation of 0.78 (95%CI: 0.40 to 0.93, p = 0.01) (figure 2A and 2B). MATV was also correlated with BF albeit with a limited statistical significance (|ρ| = 0.71, 95%CI: 0.26 to 0.90, p = 0.05) (figure 2C). In contrast, for stage III and IV, SUV_max_ was not found to be significantly correlated with BF.

Finally, none of the parameters under investigation in this study were found to be correlated with PS, BV or MTT, independently on the patient stage.

## Discussion

This study is, to our knowledge, the first of its kind assessing the correlation of recently proposed global, regional and local PET measures of intra-tumor ^18^F-FDG activity distribution heterogeneity with CT derived tumor perfusion parameters. The role and potential clinical value of tumor heterogeneity characterization is a current topic of increasing interest in ^18^F-FDG PET tumor imaging. Within this context recent studies have shown that the quantification of intra-tumor intensity voxel arrangements in ^18^F-FDG PET can provide independent prognostic and predictive factors of disease and associated treatment outcomes [9], [11], [28]. At a local level, these tumor heterogeneity parameters quantify the differences of intensity between contiguous voxels, while at the regional level they quantify relationships between homogenous areas of different intensities, associated with tumor regions of increased or reduced metabolism.

One of the remaining issues and recurrent questions is the relationship of the measured tumor ^18^F-FDG uptake heterogeneity with underlying biology. ^18^F-FDG uptake heterogeneity can be the result of many physiological processes, such as vascularization, perfusion, tumor aggressiveness, or hypoxia [29], [30]. As already mentioned, our study is the first to evaluate the correlation between PET ^18^F-FDG uptake heterogeneity patterns and CT perfusion parameters which have previously shown to reflect tumor vascularization and aggressiveness [31]. Several previous studies have in the past investigated the metabolism-perfusion correlation, comparing standard ^18^F-FDG PET image derived parameters providing a global tumor assessment (SUV_mean_, SUV_max_) and various perfusion CT parameters. These investigations have led to varying results depending on tumor type as well as tumor stage [32]. For example, Janssen et al. found that highly perfused tumors had a higher FDG uptake than relatively low perfused tumors in rectal cancer [33]. Groves et al. showed that there was a significant positive correlation between tumor SUV and tumor perfusion normalized to cardiac output for breast cancer patients [34]. Hirasawa et al. found an inverse significant correlation between tumor perfusion and glucose uptake in human head and neck tumors [35]. On the other hand, Bisdas et al. have shown a significant correlation between SUV_max_ and BF as well as between SUV_mean_ and PS for head and neck squamous cell carcinomas [36]. Miles et al found a non-consistent relationship between BF and SUVs in non-small cell lung cancer, depending on tumor size and stage, with a coupling of flow and metabolism in small tumors [37]. Finally, Goh et al. found a statistically significant correlation between tumor BF and tumor metabolism for high tumor stages (III and IV) but not for stage I or II in colorectal cancer [38].

BV and PS have been previously shown to correlate with angiogenesis in colorectal tumors [14]. In our study BV, which highlights the volume of circulating blood within the tumor vasculature, and provides a measure of ‘functional vessel density', was not correlated with SUV_max_. This result might be due to the limited number of patients included in this study. If a correlation was confirmed in a larger cohort for patients with stage I and II, this result might be explained by the fact that tracer delivery is optimal in highly vascularized small tumors leading to higher ^18^F-FDG radiotracer uptake. Also, previous studies have shown that in stage III/IV tumors, other phenomena such as hypoxia and the increased presence of necrotic regions can influence this relationship, which can explain the observed lack of significant correlation for high stages [39].

For stage I and II patients there were no statistically significant correlations between any perfusion parameters and heterogeneity parameters. The absence of any correlation with heterogeneity parameters may be due to the small size of tumors in stage I/II patients (MATV of 14±10 cm^3^) which in combination with the limited PET spatial resolution reduces the potential of local ^18^F-FDG tracer heterogeneity characterization.

On the other hand, when considering stage III/IV patients (MATV of 31±20 cm^3^), several statistically significant correlations were found between intra-tumor uptake heterogeneity features and perfusion CT parameters. These correlations concerned both local and regional intra-tumor ^18^F-FDG tracer heterogeneity parameters as well as tumor MATVs. Amongst the CT perfusion parameters considered blood flow exhibited the highest correlation with the different scale heterogeneity parameters, which suggests that ^18^F-FDG PET local and regional intra-tumor heterogeneity measurements are associated with tumor vascularization, similarly to BF.

Regarding MTT (BV/BF ratio), no statistically significant correlations were found with local or regional intra-tumor uptake heterogeneity parameters. Again the limited number of patients relative to the number of considered parameters might limit the statistical power of the study. Without correcting for multiple testing, IV, SZV and entropy were indeed correlated with MTT and BV. However if these correlations were highlighted by including more patients, they would probably remain comparatively smaller than the correlation coefficients found for BF. This can be explained by the fact that BV was not significantly correlated with the considered intra-tumor uptake heterogeneity features, leading to a weaker, according to the Spearman's rank, correlation for the ratio of BV and BF.

The choice of carrying out the analysis separately for the two subgroups was motivated by two main reasons. The first is that other phenomenon, such as hypoxia or necrotic areas, are more likely to occur in higher stage tumors. In addition, on smaller stages tumors the heterogeneity parameters are mostly correlated with MATV without providing complementary information. Dichotomization of the patients according to MATV or T stage did not modify the results obtained by dichotomizing according to AJCC stage.

One of the limitations of our study is the small number of included patients, and therefore our results need to be confirmed on a larger tumor population considering also different cancer types. A second limitation concerns the accuracy and robustness of assessing tumor perfusion using DCE-CT that is less accurate than measurements assessed with oxygen-15 water PET. Within this context it has been demonstrated that acquisition settings can have an impact on modeling methodology reproducibility and as a result on the derived perfusion parameter values [40], [41]. Although histopathological data such as VEGF, GLUT, CD105 and HiF1α could have been of interest for the present investigation, it was available for only a few patients and we could therefore not include these biomarkers in the undertaken analysis.

In this study the correlation between perfusion CT parameters and PET was investigated only using PET images with 18F-FDG that reflect tumor metabolism. Obviously as a perspective it will be interesting to investigate other radiotracers with higher specificity in terms of physiological processes targeted such as deoxy-3′-[18F]-fluorothymidine (cell proliferation) or [18F]-fluoromisonidazole (hypoxia). Since tumor heterogeneity characterization aims at quantifying such underlying physiological processes, the use of these alternative radiotracers might provide higher correlations than those obtained with FDG, whose accumulation is mediated by a combination of all these physiological processes and others (hypoxia, proliferation, vascularization, angiogenesis). Any such future study should clearly concentrate on tumor stages III and IV, since textural analysis for tumor heterogeneity characterization may be compromised for small tumors defined by a limited number of voxels in the PET images.

Finally, as a future perspective, it may be also interesting to investigate the correlation of functional and anatomical heterogeneity provided by respectively 18F-FDG PET and DCE-CT images. According to our knowledge there is no study that had investigated this kind of correlation, although anatomical DCE-CT image and FDG PET heterogeneity parameters have been both previously shown to provide valuable indicators of patient survival in non-small cell lung cancer irrespective of treatment regime [42].

## Conclusions

Statistically significant correlations were determined between BF perfusion parameter acquired on DCE-CT images and the measurement of uptake heterogeneity patterns on ^18^F-FDG PET images for stage III/IV patients. Our results confirm that intra-tumor ^18^F-FDG PET local and regional heterogeneity measures are linked to tumor vascularization as measured by DCE-CT. Intra-tumor ^18^F-FDG uptake heterogeneity patterns in the PET images may provide complementary information to perfusion parameters provided by DCE-CT.
